# Complete mitochondrial genome of *Procapra picticaudata*, with phylogenetic implication

**DOI:** 10.1080/23802359.2018.1495112

**Published:** 2019-07-12

**Authors:** Ke-Ji Guo, Hai-Jun Jiang, Feng-Jun Li, Zhi-Min Jiang, Shun-de Chen, Ge Sang, Xue-Lin Zhu, Qiong Wang, Hao Zong

**Affiliations:** a Central South Forest Inventory and Planning Institute of State Forestry Administration, Changsha, China;; b College of Life Sciences, Sichuan Normal University, Chengdu, China;; c Administration Bureau of Mt. Qomolangma National Nature Reserve, Shigatse, China;; d Tibetan Academy of Forestry Investigation and Planning, Lhasa, China

**Keywords:** *Procapra picticaudata*, complete mitochondrial genome, evolutionary relationships

## Abstract

The Tibetan gazelle *Procapra picticaudata* is endemic to the Tibetan plateau. The species is listed as a Near Threatened (NT) species by the IUCN Red List of Threatened Animals and the Red List of China’s Vertebrates. In this study, we sequenced the complete mitochondrial genome of *P. picticaudata* and examined its phylogenetic position with other nine species in Artiodactyla. The complete mitochondrial genome is 16,620 bp in length and contained 22 transfer RNA genes, 2 ribosomal RNA genes, 13 protein-coding genes, and 1 control region. Our data would provide reference information for further study of this species and be useful for evolutionary and phylogenetics studies for this NT species.

The Tibetan gazelle *Procapra picticaudata* is one of the family Bovidae. The conservation status of this species is Near Threatened (NT) in IUCN. In China, the species also has been listed as a NT species by the Red List of China’s Vertebrates (Jiang et al. 2016). The Tibetan gazelle distributes in Qinghai, Tibet, Xinjiang, and India (Jammu-Kashmir, Sikkim) and inhabits high-altitude plains, hills, wetland margins, and stony plateaux (Smith and Xie 2008). This species is one of the most geographically widespread ungulates on the Tibetan Plateau. However, the current population decline trend is due to illegal hunting, growing competition with domestic livestock, changes in land-use and government policy of fencing rangelands (Schaller 1998; Bhatnagar et al. 2006; Zhang and Jiang 2006).

We acquired some tissue of *P. picticaudata*, which killed by natural enemies in Lazi county, Xizang province, China (Latitude: 29.11°N, Longitude: 87.93°E), and maintained its tissue in Sichuan Normal University, Chengdu, Sichuan province of China. In this study, we sequenced the complete mitochondrial genome of *P. picticaudata* (Genbank number: MH345727) and examined its phylogenetic position with other nine species in Artiodactyla. Total genomic DNA was extracted from muscle tissue using the DNA extraction kit (Aidlab Biotech, Beijing, China). The mitochondrial genomes of *P. przewalskii* were used to design primers for polymerase chain reaction (PCR) and used as a template for gene annotation.

The complete mitochondrial genome of *P. picticaudata* was obtained to be 16,620 bp long and contained 22 transfer RNA genes, 2 ribosomal RNA genes, 13 protein-coding genes, and 1 control region. The overall base composition of the entire genome was as follows: A (34%), T (28.3%), C (24.7%), and G (13%), with an A + T−rich pattern of the vertebrate mitochondrial genome. As in other Artiodactyla, 12 protein-coding genes were encoded on the H-strand, with only ND6 genes encoded on the L-strand. *P. picticaudata* had one non-coding region (D-loop) located on the heavy strand between the tRNA^Pro^ and tRNA^Phe^. Total length of the 13 protein-coding genes is 11,411 bp. All of the protein-coding genes initiate with ATA or ATG start codon.

The phylogenetic relationship for *P. picticaudata* was examined with those of nine Artiodactyla species (Figure 1). Phylogenetic trees were derived from the concatenated sequence of 12 protein-coding genes by employing Bayesian inference (BI) which was performed using BEAST version 1.7.5 (Drummond et al. 2012). The best-fit GTR + I+G model was selected in jModelTest 0.1 (Darriba et al. 2012). The three *Procapra* species formed a solid monophyletic group, well supported using a Bayesian posterior probability of 1.00. Bayesian analyses also suggested that *P. picticaudata* was a sister relationship to *P. przewalskii* + *P. gutturosa* (*p* = 1.00).

**Figure 1.**
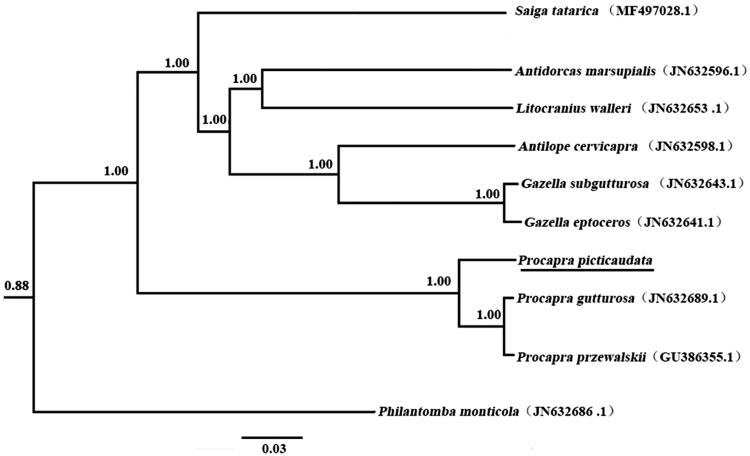
Phylogenetic tree derived from 12 protein-coding gene sequences from 10 complete mitochondrial genomes of Artiodactyla using BI analysis. Numbers by the nodes indicate Bayesian posterior probabilities.

Our data would provide reference information for further study of this species and be useful for evolutionary and phylogenetics studies for this NT species.
